# Nonlinear association and predictive value of stress hyperglycemia ratio for 28-day in-hospital mortality in patients with acute hypoxemic respiratory failure

**DOI:** 10.1097/MD.0000000000046367

**Published:** 2026-05-12

**Authors:** Zhen-ping Hu, Fang Wu, Ying-hai Zhu, Xiang-qing Xiang, Xia Zhou, Xiaohan Li, Mao Ye

**Affiliations:** a Department of Endocrinology, The Central Hospital of Enshi Tujia and Miao Autonomous Prefecture, Enshi, China; b Rehabilitation Medical Center, The Central Hospital of Enshi Tujia and Miao Autonomous Prefecture, Enshi, China; c Faculty of Medicine, Khon Kaen University, Khon Kaen, Thailand.

**Keywords:** 28-day mortality, acute hypoxemic respiratory failure, nonlinear association, prognosis, stress hyperglycemia ratio

## Abstract

Stress hyperglycemia ratio (SHR), calculated as admission glucose divided by estimated average glucose from glycated hemoglobin, has emerged as a reliable biomarker of physiological stress. However, its prognostic role in acute hypoxemic respiratory failure (AHRF) – particularly the potential nonlinear relationship with short-term mortality – remains underexplored. This study aimed to evaluate the nonlinear association and predictive value of SHR for 28-day in-hospital mortality in AHRF patients. A retrospective cohort study was conducted using data from the medical information mart for intensive care version 3.1 database (2008–2019). Patients with AHRF (ICD-10 code: J9601) were included. Propensity score matching (1:1) was applied to balance baseline characteristics (sex, age, comorbidities) between high and low SHR groups. The primary outcome was 28-day in-hospital mortality. Restricted cubic spline models explored dose–response relationships. Kaplan–Meier survival curves, multivariable Cox regression, and subgroup analyses were used to assess associations. A total of 704 patients were included, with 614 successfully matched after propensity score matching. Restricted cubic spline analysis revealed a significant nonlinear association between SHR and 28-day mortality (*P* for overall = .002; *P* for nonlinearity = .002), with a critical inflection point at 1.215. Patients with SHR > 1.215 (high SHR group) had a significantly increased mortality risk (hazard ratio [HR] = 1.508, 95% confidence interval: 1.184–1.922; log-rank *P* < .001). Subgroup analyses confirmed consistent associations across most subgroups (e.g., age, sex, diabetes), with no significant interactions (all *P* > .05). Notably, the association was amplified in patients with prior myocardial infarction or congestive heart failure (interaction *P* = .021). SHR exhibits a nonlinear association with 28-day in-hospital mortality in AHRF patients, with a critical threshold at 1.215. It serves as a simple, accessible tool for early risk stratification, particularly in those with cardiovascular comorbidities.

## 1. Introduction

Acute hypoxemic respiratory failure (AHRF) is a life-threatening intensive care unit (ICU) condition characterized by hypoxemia (PaO₂/FiO₂ < 300 mm Hg) and accounts for 30% to 40% of ICU admissions, with in-hospital mortality up to 35%.^[1,2]^ Early accurate risk stratification is critical for optimizing treatment (e.g., respiratory support escalation, resource allocation) but remains challenging due to the limitations of existing tools – starting with incomplete identification of key risk factors.^[3]^ McKown et al identified pre-intubation hypoxemia as a major predictor of hypoxemia during tracheal intubation in critically ill adults.^[4]^ This finding highlights that hypoxemia risk can be anticipated from routinely available pre-procedure data; however, because such factors often become evident only after clinical deterioration, their utility for very early risk stratification may be limited.

Conventional severity stratification indicators further face constraints: Pisani et al showed that SpO₂/FiO₂ ratio and PEEP levels at initial ARDS diagnosis and 24-hour follow-up improve risk stratification for moderate-to-severe cases,^[5]^ but these parameters are heavily dependent on ventilator settings and fail to reflect systemic stress – a key driver of AHRF outcomes. Even comprehensive management guidelines highlight this gap: Narendra et al updated recommendations for severe AHRF management,^[6]^ emphasizing the need for integrated risk assessment but not providing a simple, accessible bedside tool to bridge respiratory function and systemic stress evaluation.

In recent years, novel tools targeting specific AHRF scenarios have emerged but with narrow applicability: Zhou et al conducted a systematic review and meta-analysis confirming the respiratory oxygenation index (SpO₂/FiO₂ divided by respiratory rate) as a predictor of high-flow nasal cannula (HFNC) failure in pneumonia-related AHRF, but this index requires continuous monitoring of respiratory parameters and is less useful for non-pneumonia AHRF^[7]^; Xu et al developed a risk-stratification model for HFNC therapy in COVID-19-related AHRF, but its generalizability to non-COVID cohorts (the majority of AHRF cases) is unproven^[8]^; Li et al used machine learning for real-time prediction of HFNC failure in AHRF, but this approach demands complex data input and specialized software, making it impractical for resource-limited settings.^[9]^ These advances underscore the need for a prognostic marker that is simple, broadly applicable, and reflective of systemic stress.

Stress hyperglycemia is a key marker of systemic stress in critical illness, correlating closely with disease severity and prognosis.^[10]^ However, traditional blood glucose measurements cannot distinguish transient stress-induced hyperglycemia from chronic hyperglycemia (e.g., in diabetes), leading to inaccurate risk assessment. The stress hyperglycemia ratio (SHR) – calculated as admission glucose divided by glycated hemoglobin (HbA1c)-estimated baseline glucose – solves this by eliminating chronic glycemic interference.^[11]^ Its prognostic value has been validated in sepsis, myocardial infarction, and stroke,^[12–16]^ and study by Bai et al confirmed that elevated SHR is associated with higher hospital mortality in a broad RF cohort.^[17]^ Notably, these studies did not focus on AHRF (a distinct subtype with severe hypoxemia) nor explore potential nonlinear relationships between SHR and short-term mortality – critical gaps for AHRF-specific risk stratification.

Against this backdrop, this study aimed to evaluate the nonlinear association and predictive value of SHR for 28-day in-hospital mortality in AHRF patients using data from the medical information mart for intensive care (MIMIC-IV) database version 3.1 (2008–2019), providing a complementary tool to existing risk stratification approaches.

## 2. Materials and methods

### 2.1. Data source

This retrospective cohort study utilized data from the MIMIC-IV version 3.1 database, which contains detailed clinical information on ICU patients admitted to Beth Israel Deaconess Medical Center between 2008 and 2019. The Institutional Review Board of Beth Israel Deaconess Medical Center waived the requirement for informed consent and approved the sharing of research resources. The authors obtained access to the database (certification ID: [56073040]).

### 2.2. Study population

Inclusion criteria were: age ≥ 18 years; first ICU admission; ICD-10 code: J9601; availability of data on admission blood glucose (ABG) and HbA1c; completion of 28-day in-hospital follow-up or achievement of study endpoints during hospitalization. Exclusion criteria included repeated ICU admissions, missing key data, or an ICU stay of <24 hours.

Baseline characteristics were recorded upon ICU admission, including demographic information (age and gender), vital signs (heart rate, systolic and diastolic blood pressure, oxygen saturation, respiratory rate, and body temperature), and underlying comorbidities (myocardial infarction, congestive heart failure, cerebrovascular disease, chronic pulmonary disease, diabetes, liver disease, and renal disease). Laboratory measurements encompassed white blood cell count, platelet count, hematocrit, hemoglobin, anion gap, bicarbonate, serum levels of sodium, potassium, calcium, and chloride, as well as coagulation indicators (prothrombin time, partial thromboplastin time, international normalized ratio), renal function markers (blood urea nitrogen and serum creatinine), ABG, and HbA1c.

### 2.3. Exposure variable: SHR calculation

SHR was calculated using the formula: SHR = ABG (mg/dL)/[28.7 × HbA1c (%) −46.7]. SHR was dichotomized into high and low groups based on the inflection point identified through restricted cubic spline (RCS) analysis.

### 2.4. Outcome measures

The primary outcome was 28-day in-hospital mortality.

### 2.5. Statistical analysis

Baseline characteristics were compared between the high and low SHR groups, categorized according to the inflection point identified via RCS analysis. To ensure data quality, missing values and outliers were carefully examined and addressed using appropriate statistical methods: variables with >5% missing data were excluded from the analysis, and missing values for continuous variables were imputed using linear interpolation. Key variables (including SHR components and outcome data) had ≤5% missingness. The Kolmogorov–Smirnov test was used to assess the normality of distributions. Depending on the distribution, continuous variables were analyzed using either analysis of variance or the Kruskal–Wallis test and presented as medians with interquartile ranges (*Q*_1_–*Q*_3_). Categorical variables were expressed as frequencies and percentages (n, %) and compared using the Chi-square test. To reduce the impact of confounding factors, 1:1 propensity score matching was performed to balance baseline characteristics between groups, with matching covariates including gender, age, and underlying comorbidities (i.e., each patient in the low SHR group was matched with 1 patient in the high SHR group with similar baseline characteristics). A dose–response relationship was explored using RCS analysis to identify potential nonlinear associations between SHR and mortality. Kaplan–Meier survival curves were plotted to evaluate survival differences between SHR groups, with comparisons performed using the log-rank test. Additionally, subgroup analyses were conducted to examine the consistency of associations across various clinical subpopulations.

Statistical analyses were performed using R software (version 4.5.0), and a *P*-value < .05 was considered statistically significant.

## 3. Results

### 3.1. Baseline characteristics

A total of 704 patients with AHRF met the inclusion criteria. To reduce the interference of confounding factors such as gender, age, and comorbidities on the study results, this study used 1:1 propensity score matching to adjust patient grouping – specifically, each patient in the low SHR group was matched with 1 patient in the high SHR group with similar baseline characteristics from the original cohort. After matching, a total of 614 AHRF patients with balanced baseline characteristics were included, with 307 patients in both the low SHR group and the high SHR group. The standardized mean differences of variables between the 2 groups before and after matching are shown in Table 1, indicating that the balance of baseline characteristics between groups was significantly improved after matching.

**Table 1:** PSM before + after.

Variable	Before PSM				After PSM			
	Total (n = 704)	Low SHR (n = 352)	High SHR (n = 352)	SMD	Total (n = 614)	Low SHR (n = 307)	High SHR (n = 307)	SMD
Age, M (*Q*₁, *Q*₃)	69.1 (59.0, 78.1)	70.2 (59.0, 78.5)	68.2 (58.9, 77.4)	**0.080**	67.9 (59.8, 78.2)	65.5 (57.4, 76.0)	70.9 (64.9, 81.7)	**0.042**
Los ICU, M (*Q*₁, *Q*₃)	7.1 (2.9, 15.5)	7.8 (2.9, 16.7)	6.8 (3.0, 14.2)	**0.143**	11.0 (3.0, 15.1)	14.2 (3.8, 20.9)	6.8 (2.3, 10.3)	**0.133**
Hematocrit, M (*Q*₁, *Q*₃)	32.3 (26.7, 37.9)	33.0 (28.2, 38.2)	31.1 (25.6, 37.3)	**0.200**	32.7 (26.7, 37.9)	32.0 (26.7, 37.1)	33.6 (26.9, 39.1)	**0.139**
Hemoglobin, M (*Q*₁, *Q*₃)	10.2 (8.6, 12.2)	10.6 (8.8, 12.4)	9.9 (8.3, 12.0)	**0.156**	10.5 (8.6, 12.2)	10.3 (8.6, 11.9)	10.8 (8.6, 12.5)	**0.092**
Platelets, M (*Q*₁, *Q*₃)	192.8 (137.4, 256.1)	193.0 (141.4, 269.5)	192.2 (131.9, 248.5)	**0.121**	208.4 (140.1, 257.4)	207.6 (137.5, 266.0)	209.4 (148.0, 253.5)	**0.101**
WBC, M (*Q*₁, *Q*₃)	12.9 (9.4, 17.0)	11.2 (8.3, 15.2)	14.6 (10.7, 18.1)	**0.228**	15.1 (9.5, 17.1)	14.6 (8.7, 16.2)	15.7 (10.2, 18.0)	**0.211**
Aniongap, M (*Q*₁, *Q*₃)	14.5 (12.4, 17.5)	14.0 (12.0, 16.1)	15.0 (13.0, 19.0)	**0.357**	15.3 (12.5, 17.5)	14.6 (12.0, 16.5)	16.2 (12.5, 19.0)	**0.343**
Bicarbonate, M (*Q*₁, *Q*₃)	21.5 (18.5, 24.0)	22.0 (19.5, 24.0)	20.5 (17.5, 23.5)	**0.307**	21.3 (18.5, 24.0)	21.8 (19.0, 24.0)	20.8 (17.5, 24.0)	**0.314**
BUN, M (*Q*₁, *Q*₃)	26.5 (17.0, 43.0)	25.0 (16.5, 41.6)	27.3 (17.5, 44.6)	**0.080**	34.3 (17.5, 43.5)	31.2 (16.0, 41.0)	38.1 (19.0, 49.5)	**0.063**
Chloride, M (*Q*₁, *Q*₃)	102.0 (98.0, 106.0)	103.0 (98.5, 106.0)	101.5 (97.5, 106.0)	**0.135**	101.8 (98.0, 106.0)	101.8 (98.0, 106.0)	101.9 (98.0, 106.0)	**0.066**
Creatinine, M (*Q*₁, *Q*₃)	1.4 (1.0, 2.2)	1.3 (0.9, 2.1)	1.4 (1.0, 2.4)	**0.026**	1.9 (1.0, 2.2)	1.9 (0.9, 2.1)	2.0 (1.0, 2.5)	**0.025**
ABG, M (*Q*₁, *Q*₃)	152.0 (123.0, 198.0)	127.8 (109.4, 151.1)	185.5 (153.8, 238.3)	**0.701**	176.2 (124.0, 197.5)	170.9 (122.0, 191.5)	182.9 (129.5, 213.5)	**0.663**
Sodium, M (*Q*₁, *Q*₃)	138.0 (134.5, 141.5)	138.5 (135.0, 141.5)	137.5 (134.0, 141.0)	**0.151**	138.0 (134.5, 141.5)	137.7 (135.0, 141.5)	138.3 (134.0, 141.5)	**0.097**
HbA1C, M (*Q*₁, *Q*₃)	5.9 (5.4, 7.0)	6.1 (5.5, 7.4)	5.8 (5.2, 6.6)	**0.422**	6.6 (5.4, 7.0)	6.6 (5.4, 7.1)	6.5 (5.4, 7.0)	**0.422**
Calcium, M (*Q*₁, *Q*₃)	8.5 (8.0, 8.9)	8.5 (8.1, 9.0)	8.4 (8.0, 8.8)	**0.078**	8.4 (8.0, 8.9)	8.4 (7.9, 8.9)	8.5 (8.1, 8.9)	**0.077**
INR, M (*Q*₁, *Q*₃)	1.4 (1.2, 1.7)	1.4 (1.2, 1.6)	1.4 (1.2, 1.8)	**0.187**	1.6 (1.2, 1.7)	1.5 (1.2, 1.6)	1.7 (1.2, 1.8)	**0.129**
PT, M (*Q*₁, *Q*₃)	15.0 (12.9, 17.8)	15.0 (12.9, 17.5)	15.0 (13.0, 19.2)	**0.164**	17.4 (13.0, 17.7)	16.8 (13.1, 17.4)	18.2 (12.8, 19.0)	**0.091**
PTT, M (*Q*₁, *Q*₃)	33.5 (28.3, 49.3)	32.8 (28.1, 45.4)	34.5 (28.4, 51.1)	**0.111**	43.0 (28.3, 49.0)	40.9 (27.8, 45.3)	45.7 (28.9, 54.2)	**0.072**
Heart rate, M (*Q*₁, *Q*₃)	85.7 (74.9, 98.7)	85.2 (73.7, 98.0)	86.5 (76.1, 99.8)	**0.072**	87.8 (74.9, 98.7)	87.4 (75.2, 97.6)	88.3 (74.3, 100.5)	**0.032**
SBP, M (*Q*₁, *Q*₃)	114.0 (104.2, 127.2)	116.5 (104.8, 129.7)	111.9 (104.1, 124.7)	**0.190**	116.9 (104.8, 126.8)	117.5 (105.5, 126.7)	116.1 (103.2, 126.8)	**0.139**
DBP, M (*Q*₁, *Q*₃)	62.4 (56.3, 69.2)	63.0 (56.5, 70.8)	61.6 (55.7, 68.1)	**0.149**	63.2 (56.0, 69.5)	63.7 (57.1, 70.8)	62.5 (55.3, 68.2)	**0.119**
Respiratory rate, M (*Q*₁, *Q*₃)	20.5 (18.0, 23.1)	20.1 (17.7, 22.7)	20.7 (18.2, 23.3)	**0.127**	20.9 (18.0, 23.3)	20.3 (17.8, 22.3)	21.6 (18.7, 24.0)	**0.165**
Temperature, M (*Q*₁, *Q*₃)	36.9 (36.7, 37.2)	36.9 (36.7, 37.3)	36.9 (36.6, 37.1)	**0.244**	37.0 (36.7, 37.3)	37.0 (36.7, 37.3)	37.0 (36.6, 37.3)	**0.273**
Spo_2_, M (*Q*₁, *Q*₃)	96.7 (95.2, 98.2)	96.5 (95.1, 97.9)	97.0 (95.2, 98.4)	**0.126**	96.6 (95.2, 98.2)	96.8 (95.6, 98.2)	96.2 (94.7, 98.2)	**0.176**
Male, n (%)	445 (63.2%)	230 (65.3%)	215 (61.1%)	**0.088**	399 (65.0%)	226 (65.5%)	173 (64.3%)	**0.020**
Myocardial infarct, n (%)	209 (29.7%)	97 (27.6%)	112 (31.8%)	**0.093**	190 (30.9%)	94 (27.2%)	96 (35.7%)	**0.042**
Congestive heart failure, n (%)	327 (46.5%)	174 (49.4%)	153 (43.5%)	**0.120**	295 (48.1%)	161 (46.7%)	134 (49.8%)	**0.046**
Cerebrovascular disease, n (%)	286 (40.6%)	160 (45.5%)	126 (35.8%)	**0.197**	242 (39.4%)	116 (33.6%)	126 (46.8%)	**0.013**
Chronic pulmonary disease, n (%)	160 (22.7%)	91 (25.9%)	69 (19.6%)	**0.149**	134 (21.8%)	68 (19.7%)	66 (24.5%)	**0.063**
Renal disease, n (%)	245 (34.8%)	126 (35.8%)	119 (33.8%)	**0.042**	215 (35.0%)	123 (35.7%)	92 (34.2%)	**0.034**
Diabetes, n (%)	359 (51.0%)	179 (50.9%)	180 (51.1%)	**0.006**	326 (53.1%)	187 (54.2%)	139 (51.7%)	**0.013**
Liver disease, n (%)	121 (17.2%)	46 (13.1%)	75 (21.3%)	**0.219**	93 (15.2%)	56 (16.2%)	37 (13.8%)	**0.009**

The significance of the bolded values (standardized mean difference [SMD]) is defined based on the criteria for assessing baseline balance between groups: an absolute SMD value ≥0.1 indicates a meaningful imbalance in the corresponding covariate between the 2 groups, suggesting a potential confounding bias that requires attention; and an absolute SMD value <0.1 indicates good balance of the covariate between groups, with effective control of confounding bias.

ABG = admission blood glucose, BUN = blood urea nitrogen, DBP = diastolic pressure, HbA1c = glycosylated hemoglobin, INR = international normalized ratio, PLT = platelet count, PT = prothrombin time, PTT = partial thromboplastin time, SBP = systolic blood pressure, SHR = stress hyperglycemia ratio, SMD = standardized mean difference, SpO_2_ = pulse oxygen saturation, WBC = white blood cell.

### 3.2. Restricted cubic spline analysis based on cox regression

**Figure 1.**
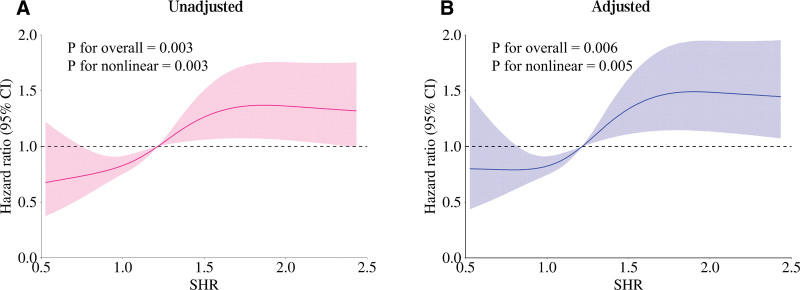
Restricted cubic spline (RCS) plots showing the nonlinear association between stress hyperglycemia ratio (SHR) and 28-d in-hospital mortality in patients with acute hypoxemic respiratory failure (AHRF). (A) Unadjusted model: overall association *P* = .003, nonlinearity *P* = .003. (B) Adjusted model (adjusted for gender, age, comorbidities, and vital signs): overall association *P* = .006, nonlinearity *P* = .005. The *x*-axis represents SHR values, and the *y*-axis represents the hazard ratio (HR) for mortality 1. AHRF = acute hypoxemic respiratory failure, RCS = restricted cubic splines, SHR = stress hyperglycemia ratio.

RCS functions were incorporated into the Cox proportional hazards model to assess the nonlinear association between SHR and 28-day in-hospital mortality. In the unadjusted model, a significant overall association was observed (*P* for overall = .002), with evidence of a nonlinear relationship (*P* for nonlinearity = .004; Fig. 1A). After adjusting for gender, age, comorbidities, and vital signs, the nonlinear association remained statistically significant (*P* for overall = .002; *P* for nonlinearity = .002; Fig. 1B). An inflection point was identified at an SHR value of 1.215, which was subsequently used to dichotomize patients into low and high SHR groups for further analysis.

Based on the inflection point identified through dose–response RCS analysis, patients were divided into low SHR (≤1.215) and high SHR (>1.215) groups, each accounting for 50% of the matched cohort. Statistical analysis revealed significant differences between the 2 groups in white blood cell, anion gap, bicarbonate, glucose, HbA1c, respiratory rate, SpO_2_, and 28-day in-hospital mortality (all *P* < .05; detailed demographic and clinical characteristics are shown in Table 2).

**Table 2:** Baseline characteristics of patients with acute hypoxemic respiratory failure.

Variables	Total (n = 614)	SHR ≤ 1.215 (n = 307)	SHR>1.215 (n = 307)	Statistic	*P*
Demographics					
Age, M (*Q*₁, *Q*₃)	69.3 (59.8, 78.2)	69.3 (58.3, 78.3)	69.4 (60.6, 78.0)	−0.18	.856
Male, n (%)	399 (64.98)	201 (65.47)	198 (64.50)	0.06	.800
Laboratory examination, M (*Q*₁, *Q*₃)					
Hematocrit, %	32.5 (26.7, 37.9)	32.7 (28.1, 37.9)	31.5 (25.7, 37.7)	−1.68	.092
Hemoglobin, g/dL	10.3 (8.6, 12.2)	10.4 (8.7, 12.2)	10.1 (8.4, 12.1)	−1.35	.177
Platelets, 10^9^/L	194.3 (140.1, 257.4)	193.5 (143.0, 271.5)	196.5 (138.3, 250.0)	−0.69	.493
WBC, 10^9^/L	12.9 (9.5, 17.1)	11.4 (8.2, 15.4)	14.3 (10.7, 18.1)	−5.57	**<.001**
Aniongap, mmol/L	14.5 (12.5, 17.5)	14.0 (12.0, 16.5)	15.0 (13.0, 19.0)	−3.91	**<.001**
Bicarbonate, mmol/L	21.5 (18.5, 24.0)	22.0 (19.5, 24.0)	20.5 (17.5, 23.5)	−3.78	**<.001**
BUN, mg/dL	27.0 (17.5, 43.5)	26.5 (17.0, 42.5)	27.5 (18.0, 44.3)	−1.04	.296
Chloride, mmol/L	102.0 (98.0, 106.0)	103.0 (98.0, 106.0)	102.0 (97.5, 106.0)	−0.69	.487
Creatinine, mg/dL	1.4 (1.0, 2.3)	1.4 (0.9, 2.2)	1.4 (1.0, 2.4)	−1.26	.207
ABG, mg/dL	154.0 (124.0, 197.5)	130.0 (110.0, 154.8)	186.0 (154.0, 238.0)	−13.48	**<.001**
Sodium, mmol/L	138.0 (134.5, 141.5)	138.0 (135.0, 141.5)	138.0 (134.0, 141.0)	−1.13	.259
HbA1C, %	6.0 (5.4, 7.0)	6.1 (5.5, 7.7)	5.8 (5.2, 6.7)	−4.13	**<.001**
Calcium, mmol/L	8.4 (8.0, 8.9)	8.5 (8.0, 9.0)	8.4 (7.9, 8.8)	−1.35	.176
INR, s	1.4 (1.2, 1.7)	1.4 (1.2, 1.6)	1.4 (1.2, 1.7)	−0.16	.871
PT, s	14.9 (13.0, 17.7)	15.2 (13.0, 17.5)	14.8 (12.9, 18.6)	−0.12	.908
PTT, s	33.1 (28.3, 49.0)	32.9 (28.1, 45.9)	33.2 (28.4, 52.2)	−0.49	.622
Vital signs, M (*Q*₁, *Q*₃)					
Heart rate, beats per minute	86.4 (74.9, 98.7)	86.4 (74.3, 98.3)	86.4 (75.2, 99.7)	−0.57	.571
SBP, mm Hg	114.0 (104.8, 126.8)	116.2 (104.8, 128.8)	112.9 (104.8, 125.5)	−1.67	.095
DBP, mm Hg	62.4 (56.0, 69.5)	62.9 (56.4, 70.8)	62.1 (55.8, 68.4)	−1.43	.153
Respiratory rate, breathes per minute	20.5 (18.0, 23.3)	20.1 (17.7, 23.1)	20.9 (18.3, 23.7)	−1.97	**.048**
Temperature, ℃	36.9 (36.7, 37.3)	37.0 (36.7, 37.3)	36.9 (36.6, 37.2)	−2.96	**.003**
SpO_2_, %	96.7 (95.2, 98.2)	96.5 (95.1, 97.8)	97.1 (95.5, 98.4)	−2.66	**.008**
Outcome					
Los ICU, M (*Q*₁, *Q*₃), h	7.1 (3.0, 15.1)	7.8 (2.9, 15.7)	6.9 (3.1, 14.3)	−0.65	.514
Hospital expire flag, n (%)	269 (43.8)	115 (37.5)	154 (50.2)	10.06	**.002**
Comorbidities, n (%)					
Myocardial infarct	190 (30.9)	92 (30.0)	98 (31.9)	0.27	.600
Congestive heart failure	295 (48.1)	151 (49.2)	144 (46.9)	0.32	.572
Cerebrovascular disease	242 (39.4)	122 (39.7)	120 (39.1)	0.03	.869
Chronic pulmonary disease	134 (21.8)	71 (23.1)	63 (20.5)	0.61	.434
Renal disease	215 (35.0)	105 (34.2)	110 (35.8)	0.18	.672
Diabetes	326 (53.1)	164 (53.4)	162 (52.8)	0.03	.872
Liver disease	93 (15.2)	46 (15.0)	47 (15.3)	0.01	.910

Z: Mann–Whitney test, χ²: Chi-square test, M: median, *Q*₁: first quartile, *Q*₃: third quartile.

ABG = admission blood glucose, BUN = blood urea nitrogen, DBP = diastolic pressure, HbA1c = glycosylated hemoglobin, INR = international normalized ratio, PLT = platelet count, PT = prothrombin time, PTT = partial thromboplastin time, SBP = systolic blood pressure, SHR = stress hyperglycemia ratio, SpO_2_ = pulse oxygen saturation, WBC = white blood cell.

### 3.3. Kaplan–Meier analysis

**Figure 2.**
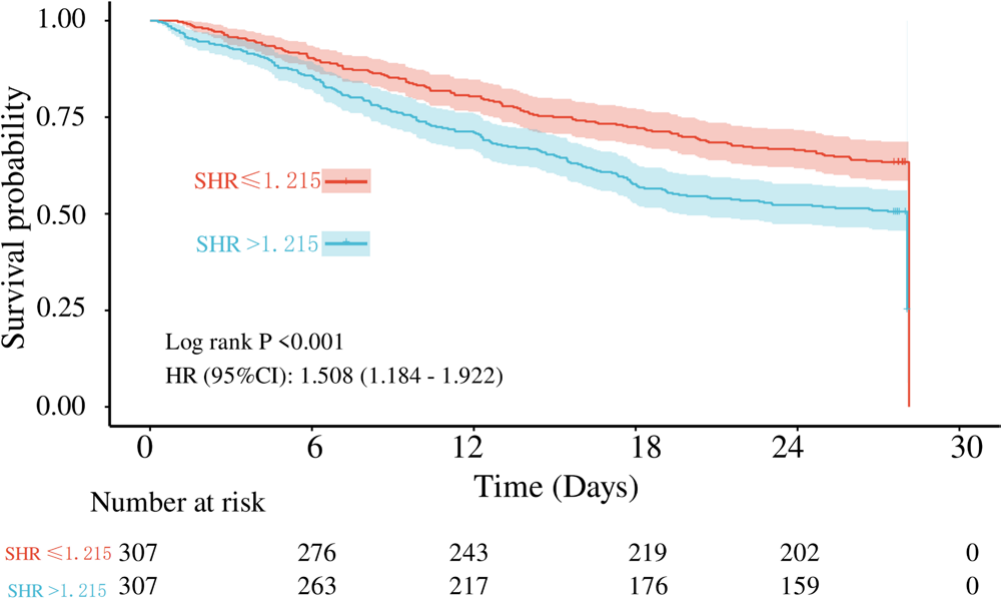
Kaplan–Meier survival curves comparing 28-day in-hospital survival between low SHR (≤1.215) and high SHR (>1.215) groups in AHRF patients after propensity score matching. The log-rank test showed a significant difference in survival (*P* < .001). The HR for mortality in the high SHR group was 1.508 (95% CI: 1.184–1.922). The table below the curve shows the number of patients at risk in each group at specified time points (d). AHRF = acute hypoxemic respiratory failure, SHR = stress hyperglycemia ratio.

Kaplan–Meier survival analysis demonstrated that patients in the high SHR group had significantly lower 28-day survival compared to those in the low SHR group (log-rank *P* < .001). The HR for mortality in the high SHR group was 1.508 (95% confidence interval [CI]: 1.184–1.922), indicating a significantly increased risk of death (Fig. 2).

### 3.4. Subgroup analyses

To explore whether the relationship between SHR and 28-day mortality varied among different comorbidity groups, subgroup analyses were conducted using multivariable Cox regression models. In the unadjusted analysis for all patients, the HR (95% CI) was 1.51 (1.18–1.92) with *P* < .001 and a *P* for interaction of .068. After adjusting for potential confounders (gender, age, comorbidities, vital signs), the HR (95% CI) for all patients was 1.48 (1.15–1.90) with *P* = .002 and a *P* for interaction of .091.

Notably, in patients with a history of myocardial infarction (adjusted HR = 2.30, 95% CI: 1.47–3.59, *P* < .001) and congestive heart failure (adjusted HR = 2.22, 95% CI: 1.54–3.20, *P* < .001), high SHR was associated with a markedly increased risk of death. An overall significant interaction was observed across these 2 subgroups (*P* for interaction = .021), indicating a potential amplifying effect of myocardial infarction and congestive heart failure on the SHR-mortality relationship.

**Figure 3.**
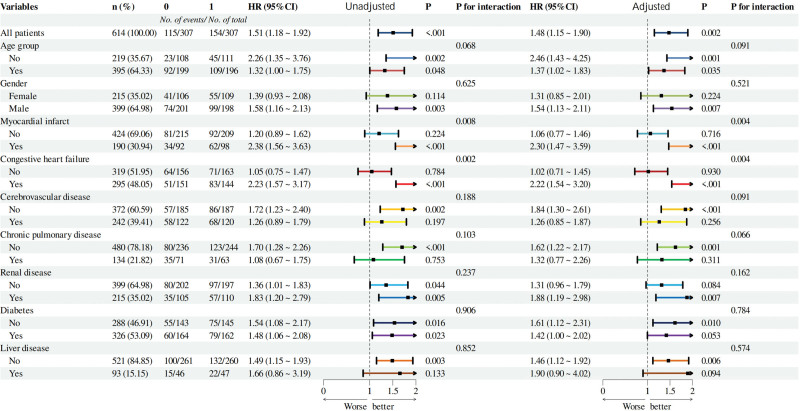
Subgroup analyses of the association between SHR and 28-d in-hospital mortality in AHRF patients. AHRF = acute hypoxemic respiratory failure, CI = confidence interval, HR = hazard ratio, SHR = stress hyperglycemia ratio.

In contrast, among patients with or without diabetes, the association between SHR and mortality remained consistent (HR = 1.48, 95% CI: 1.06–2.08 in non-diabetics; HR = 1.54, 95% CI: 1.08–2.17 in diabetics), with no significant interaction (*P* for interaction = .634), suggesting the effect of SHR on prognosis was similar regardless of diabetes status (Fig. 3).

## 4. Discussion

This study, using RCS analysis combined with Cox regression, is the first to identify a significant nonlinear association between the SHR and 28-day in-hospital mortality in patients with AHRF. Both unadjusted and adjusted models demonstrated highly significant nonlinear relationships (unadjusted: overall *P* = .002, nonlinearity *P* = .004; adjusted: overall *P* = .002, nonlinearity *P* = .002), indicating that the impact of SHR on prognosis is not linear but increases sharply beyond a threshold of 1.215. This highlights the clinical importance of this cutoff for risk stratification in AHRF. Similar nonlinear relationships between SHR and prognosis have been reported in various critical illnesses, supporting the generalizability of this phenomenon.^[13,18,19]^

Kaplan–Meier survival analysis further confirmed that patients in the high SHR group had significantly poorer 28-day survival compared to the low SHR group (log-rank *P* < .001), with a markedly increased mortality risk (HR = 1.508, 95% CI: 1.184–1.922). These findings are consistent with prior studies demonstrating SHR as an independent prognostic marker in sepsis,^[13,20,21]^ myocardial infarction,^[14,22–24]^ stroke,^[15,25–27]^ and heart failure.^[16,28–30]^ By extending this evidence to AHRF, our study fills a critical gap in understanding SHR’s prognostic value in this high-risk population, supporting its role as a practical tool for early risk stratification.

Subgroup analyses revealed that the association between SHR and 28-day mortality remained consistent across diverse clinical subpopulations. Factors such as age, sex, diabetes, liver disease, and renal disease did not significantly modify this relationship, reinforcing SHR’s utility as an independent prognostic marker. Notably, SHR exhibited significantly stronger predictive performance in patients with prior myocardial infarction and congestive heart failure (interaction *P* < .01), suggesting heightened sensitivity to stress hyperglycemia in this cardiovascular comorbidity subgroup.

Our findings – both the SHR-mortality association in AHRF and its amplified effect in heart failure patients – align with and extend the work of Bai et al^[17]^ who evaluated SHR’s prognostic role in a broad cohort of respiratory failure (RF) patients using the MIMIC-IV 3.0 database. Bai et al reported 2 key observations that resonate with our results: first, elevated SHR was independently associated with higher in-hospital mortality in RF patients (HR for *Q*_4_ vs *Q*_1_: 4.58, 95% CI: 1.83–11.5 in their unadjusted model), confirming SHR’s utility as a prognostic marker for RF-related mortality; second, they identified a U-shaped nonlinear relationship between SHR and in-hospital/ICU mortality, which supports our finding of a threshold effect (SHR = 1.215) in AHRF.

This amplified SHR-mortality association in heart failure patients may be attributed to heart failure–induced tissue hypoperfusion and metabolic disturbances, which amplify the deleterious effects of hyperglycemia, exacerbate myocardial workload and systemic inflammation,^[31–34]^ and ultimately worsen prognosis. Clinically, intensified glucose monitoring and control in heart failure patients, alongside active intervention against stress hyperglycemia, may improve outcomes.

Similarly, the relationship between SHR and mortality was particularly pronounced in patients with heart failure, whereas no significant association was observed in those without heart failure. This finding suggests that heart failure–induced tissue hypoperfusion and metabolic disturbances may amplify the deleterious effects of hyperglycemia, exacerbating myocardial workload and systemic inflammation,^[35,36]^ and ultimately worsening prognosis. Clinically, intensified glucose monitoring and control in heart failure patients, alongside active intervention against stress hyperglycemia, may improve outcomes.

Notably, the consistency of SHR’s prognostic value across diabetic and nondiabetic patients supports its utility in distinguishing acute stress hyperglycemia from chronic glycemic dysregulation – even in patients with preexisting diabetes, where isolated glucose measurements may be less reliable due to baseline glycemic variability.

Our findings highlight the incremental prognostic value of SHR in specific high-risk cardiovascular cohorts, providing valuable insights for clinical risk stratification and personalized therapeutic strategies. Future research should further explore interventions targeting stress hyperglycemia modulation to improve outcomes in these vulnerable populations.

However, several limitations should be acknowledged. This was a retrospective study using data from the MIMIC-IV database, which may introduce selection bias and limit causal inference. Additionally, residual confounding may persist despite multivariable adjustment, and we lacked detailed data on specific interventions (e.g., insulin therapy protocols, duration of hyperglycemia) that could influence outcomes. Generalizability may also be limited by the single-center nature of the database. Prospective, multicenter studies are warranted to validate these findings.

In conclusion, SHR provides an effective risk stratification tool for AHRF patients, revealing a complex nonlinear relationship with mortality risk. Integrating SHR into clinical practice to guide dynamic glucose management may improve patient outcomes. Further multicenter prospective studies and randomized controlled trials are needed to validate SHR’s utility in critical care settings.

## 5. Conclusion

In this retrospective cohort study of patients with AHRF, we identified that the SHR serves as a valuable prognostic marker for 28-day in-hospital mortality. Through RCS analysis, we confirmed a significant nonlinear association between SHR and mortality, with a critical inflection point at 1.215. Beyond this threshold, patients in the high SHR group (>1.215) exhibited a 52.9% increased risk of death compared to those in the low SHR group (≤1.215), as validated by Kaplan–Meier survival analysis.

Subgroup analyses further revealed that the prognostic value of SHR was consistent across most clinical populations, including patients with and without diabetes. Notably, the association between elevated SHR and mortality was amplified in patients with preexisting myocardial infarction or congestive heart failure, highlighting the particular vulnerability of cardiovascular comorbid patients to the deleterious effects of stress hyperglycemia.

These findings support the clinical utility of SHR as an accessible, cost-effective tool for early risk stratification in AHRF. By distinguishing acute stress-induced hyperglycemia from chronic glycemic dysregulation, SHR addresses a key limitation of traditional glucose measurements and enables targeted risk assessment.

In summary, SHR ≥ 1.215 independently predicts poor 28-day outcomes in AHRF, with heightened significance in patients with underlying cardiovascular disease. Integrating SHR into routine clinical practice may facilitate personalized risk management and guide timely interventions to improve survival in this high-risk population. Prospective multicenter studies and interventional trials are warranted to further validate these findings and explore the impact of SHR-guided glycemic management strategies.

## Author contributions

**Conceptualization**: Zhen-ping Hu, Mao Ye.

**Data curation**: Zhen-ping Hu, Fang Wu, Ying-hai Zhu, Mao Ye.

**Formal analysis**: Zhen-ping Hu, Fang Wu, Ying-hai Zhu, Xiang-qing Xiang, Xiaohan Li, Mao Ye.

**Funding acquisition**: Zhen-ping Hu, Fang Wu, Ying-hai Zhu, Xiang-qing Xiang, Xia Zhou, Xiaohan Li, Mao Ye.

**Investigation**: Zhen-ping Hu, Fang Wu, Ying-hai Zhu, Xiang-qing Xiang, Xiaohan Li, Mao Ye.

**Methodology**: Zhen-ping Hu, Fang Wu, Xia Zhou, Xiaohan Li, Mao Ye.

**Project administration**: Zhen-ping Hu, Fang Wu, Xiaohan Li, Mao Ye.

**Resources**: Zhen-ping Hu, Fang Wu, Ying-hai Zhu, Xiang-qing Xiang, Xia Zhou, Xiaohan Li, Mao Ye.

**Software**: Zhen-ping Hu, Fang Wu, Ying-hai Zhu, Xiaohan Li, Mao Ye.

**Supervision**: Zhen-ping Hu, Fang Wu, Xia Zhou, Xiaohan Li, Mao Ye.

**Validation**: Zhen-ping Hu, Fang Wu, Ying-hai Zhu, Xiang-qing Xiang, Xiaohan Li, Mao Ye.

**Visualization**: Zhen-ping Hu, Fang Wu, Ying-hai Zhu, Xiang-qing Xiang, Xia Zhou, Xiaohan Li, Mao Ye.

**Writing – original draft**: Zhen-ping Hu, Fang Wu, Mao Ye.

**Writing – review & editing**: Zhen-ping Hu, Fang Wu, Mao Ye.
